# Iatrogenic Amyloid Polyneuropathy Following Domino Liver Transplantation: A Case Report

**DOI:** 10.7759/cureus.53605

**Published:** 2024-02-05

**Authors:** Bárbara Junqueira, Carlos Mestre

**Affiliations:** 1 Family Health Unit Cartaxo Terra Viva, Regional Health Administration of Lisbon and Tagus Valley, Cartaxo, PRT

**Keywords:** iatrogenic polyneuropathy, transthyretin amyloidosis, domino liver transplantation, acquired amyloid neuropathy, familial amyloid neuropathy

## Abstract

Familial amyloidotic polyneuropathy (FAP) is an autosomal dominant hereditary disorder. In Portugal, it is mainly linked to transthyretin (TTR) mutation, and patients present with length-dependent sensory-motor polyneuropathy, often accompanied by autonomic dysfunction. Treatment options for FAP include liver transplant, and due to the lack of organs, FAP livers began being implanted in patients with severe liver disease in a process known as domino liver transplantation (DLT). We report a case of a 68-year-old Portuguese man, with post-hepatitis C-related cirrhosis liver transplantation, who presented to his family doctor with decreased sensitivity in both feet and weight loss, which were initially attributed to diabetic neuropathy and an adjustment in diabetic medication, respectively. Symptoms evolved to changes in both feet's thermal and painful sensitivity, reduced sensitivity in both hands, diarrhea, and progressive weight loss. At this time, the patient's disclosure of receiving a DLT prompted the correct diagnosis of iatrogenic amyloid polyneuropathy. This case underscores the challenges in diagnosing and managing iatrogenic amyloid polyneuropathy following DLT, highlighting the importance of prompt identification of DLT recipients, active vigilance of these patients via structured monitoring, and increased healthcare providers' awareness of this practice so that early signs of the disease may be recognized.

## Introduction

Familial amyloidotic polyneuropathy (FAP) is an autosomal dominant hereditary disease characterized by the deposition of amyloid fibers in tissues, primarily attributed to mutations in transthyretin (TTR) [1]. Alternatively, patients may present mutations in apolipoprotein A1 or gelsolin, albeit less frequently. The different types of FAP vary in their presentation and require different approaches to patient management [2].

In Portugal, most cases are associated with the presence of the mutant TTR, with the Val30Met mutation being the most common, and the first neurological symptoms usually occur in adult patients in their mid-30s [2]. TTR FAP typically causes a length-dependent sensory-motor polyneuropathy that starts in the feet with a loss of temperature and pain sensations, followed by impaired light touch sensation and motor deficits. At this stage, the patient may complain of difficulty walking and present with loss of balance and a steppage gait. These symptoms extend proximally and eventually also start affecting the upper limbs. Patients also manifest autonomic dysfunction during this phase, commonly involving the cardio-circulatory, gastrointestinal, and genitourinary systems. Some of the most common symptoms caused by autonomic dysfunction include orthostatic hypotension, episodic postprandial diarrhea, or severe constipation. In men, erectile dysfunction is an early feature that might precede sensory symptoms of neuropathy. Urinary symptoms, including dysuria and urinary retention, occur later. Some extra-neurological manifestations are cardiac involvement, reported in about 80% of cases, and, more rarely, ocular manifestations, renal involvement, and weight loss [2,3].

TTR is mainly synthesized by the liver, and liver transplantation is the cornerstone of TTR FAP treatment [4-6]. Due to the scarcity of available livers for transplantation and the fact that despite the production of mutant TTR, the liver of a patient with FAP maintains normal structure and function, livers from FAP patients that are explanted are implanted into patients with severe liver disease in a process called sequential transplantation or domino liver transplantation (DLT) [2,6]. Portugal has the most DLTs reported to the Domino Liver Transplant Registry. By December 31, 2019, 591 DLTs were reported, some of them including livers provided by FAP patients [7].

Although initially underestimated, it seems that up to 8%-24% of DLT recipients may develop iatrogenic amyloid polyneuropathy, with symptoms occurring after a median of seven years (5.7 ± 3.2 years, ranging from two months to 10 years) [8-10]. The progression of symptoms may justify a retransplantation, which has been reported to stabilize or improve neuropathy in some recipients [11,12].

## Case presentation

We present a case of a 68-year-old Portuguese retired man (former retail store manager) with a personal medical history of liver transplantation due to hepatitis C-related cirrhosis and hepatocellular carcinoma. He also had type 2 diabetes mellitus, hypertension, and dyslipidemia. The patient underwent incisional hernia repair and inguinal and umbilical hernia repairs later on. He takes metformin 860 mg twice daily, sitagliptin 25 mg once daily, insulin 10 units once daily, perindopril 4 mg once daily, simvastatin 20 mg once daily, sirolimus 1 mg once daily, and ursodeoxycholic acid 250 mg twice daily. There was no significant family medical history. About ten years post-liver transplantation, the patient's family doctor observed weight loss following an increase in metformin dosage and the discontinuation of insulin. The patient reported no other complaints, and comprehensive blood work, including thyroid function tests, was conducted. The results, detailed in Table 1, revealed no significant alterations. Therefore, it was inferred that the weight loss could be attributed to the medication switch.

**Table 1:** Patient's laboratory tests

	Results	Reference range
Complete blood count		
Erythrocytes	4.86	4.3 - 5.9 × 10^6^/mm^3^
Hemoglobin	14.5	13.5 - 17.5 g/dL
Hematocrit	44.8	41 - 53%
Mean corpuscular volume	92.2	80 - 100 fL
Mean corpuscular hemoglobin	29.8	25 - 35 pg
Mean corpuscular hemoglobin concentration	32.4	31 - 36 g/dL
Leucocytes	8.17	4.5 - 11 × 10^3^/mm^3^
Neutrophils	69.5	54 - 62%
Eosinophils	0.4	1 - 3%
Basophils	0.1	0 - 0.75%
Lymphocytes	21.9	25 - 33%
Monocytes	8.1	3 - 7%
Platelets	217	150 - 400 × 10^3^/mm^3^
Red blood cell distribution width	14.2	11.5 - 14.5%
Comprehensive metabolic panel		
A1c hemoglobin	6	<6.5%
Creatinine	1	0.6 - 1.2 mg/dL
Aspartate aminotransferase	32	15 - 40 U/L
Alanine transaminase	39	10 - 40 U/L
Gamma-glutamyl transpeptidase	119	8 - 78 U/L
Total bilirubin	0.3	0.1 - 1.0 mg/dL
Sodium	142	135 - 146 mmol/L
Potassium	4.4	3.5 - 5.0 mmol/L
Chloride	104	95 - 105 mmol/L
Ferritin	194	15 - 200 ng/mL
Folic acid	13	>5.4 ng/mL
B12 vitamin	285	190 - 950 pg/mL
Thyroid-stimulating hormone	1.6	0.5 - 5.0 mU/L

A year later, the patient complained of changes in thermal and painful sensitivity in both feet. He also began experiencing diarrhea and maintained weight loss (10 kg in one year, with 5 kg lost in the previous six months). Upon physical examination, the patient appeared in good overall condition with no evident abdominal abnormalities. Normal bowel sounds and a non-tender abdomen were noted upon palpation. A subtle steppage gait and altered thermal and painful sensitivity in both feet were observed. No other abnormalities were detected in the neurological examination. Initially, considering diabetic neuropathy as the main diagnostic hypothesis, lower limb electromyography was requested, and the patient is currently awaiting its scheduling. It was only during this consultation that the patient disclosed to his family doctor that he had received a DLT from a donor with FAP, prompting the family doctor to advise him to report the complaints in his liver transplant follow-up appointment at the hospital where he receives his follow-up care.

At the hospital, a salivary gland biopsy was performed, revealing amyloid deposition, confirmed with Congo red staining. Therefore, the diagnosis of acquired amyloid polyneuropathy following transplantation with a diseased liver was established, prompting plans to proceed with liver retransplantation.

One year after the definitive diagnosis, the patient maintained weight loss, worsening gait disturbances, and episodes of diarrhea, along with complaints of urinary incontinence that had been present for four months. Due to disease progression, he was referred to cardiology for pre-transplant evaluation.

## Discussion

This case posed several challenges. The initial neurological complaints were mistakenly attributed to potential diabetic neuropathy due to the unknown DLT. Additionally, the weight loss, initially linked to changes in diabetes medication, might have been an early sign of iatrogenic amyloid polyneuropathy.

The initial consideration of the amyloid polyneuropathy diagnosis was overlooked because, in Portugal, the typical onset of symptoms occurs in the fourth decade of life, and patients commonly have other family members with the disease, which facilitates the diagnosis [2]. Consequently, alternative diagnoses were explored for the patient's complaints. It was only when the patient disclosed receiving a liver from an individual with FAP that this hypothesis was taken into consideration.

DLT emerged as a solution to address the organ scarcity for transplantation. Despite being the country with the most reported DLTs, many Portuguese primary care providers must be made aware of this procedure. Enhancing awareness about DLTs among healthcare professionals is crucial for timely recognition and management of potential complications in recipients. Moreover, recipients of DLT should be meticulously identified to facilitate the swift recognition of any disease symptoms. Both patients and primary care providers must be well-informed and attentive to early signs of neurological symptoms post-transplant. This heightened awareness can lead to earlier interventions and improved outcomes. Implementing active surveillance for signs or symptoms in DLT recipients would also ensure early retransplantation, if necessary. Early detection and intervention may significantly impact the course of the disease, emphasizing the importance of establishing structured monitoring protocols for this specific population.

The uncertainty surrounding the progression of iatrogenic amyloid polyneuropathy post-transplant adds a layer of complexity to patient management. In some recipients, retransplantation has been reported to stabilize or improve neuropathy, highlighting the importance of considering early retransplantation. Despite liver retransplantation typically carrying a worse prognosis and more significant morbidity than the initial transplantation, survival after liver retransplantation in DLT recipients seems to be greater than in other subgroups of liver retransplant recipients [13]. This approach necessitates careful evaluation and personalized decision-making for each patient to optimize outcomes.

The complex interplay of diagnostic challenges, awareness issues, and uncertainties in iatrogenic amyloid polyneuropathy necessitates a comprehensive and informed approach to patient management, emphasizing ongoing research, awareness campaigns, and personalized care strategies. The absence of a DLT mentioned in the medical records available to the family doctor further complicated the identification of a crucial differential diagnosis.

## Conclusions

In conclusion, this case underscores the complexity of managing iatrogenic amyloid polyneuropathy post-liver transplantation. The initial misattribution of symptoms to diabetic neuropathy and delayed consideration of amyloid polyneuropathy underscore the need for heightened awareness among healthcare professionals. The emergence of DLT necessitates increased awareness among primary care providers to recognize and manage complications promptly, emphasizing the importance of DLT being mentioned in medical records. The potential benefits of retransplantation in stabilizing or improving neuropathy, along with increased risk and morbidity after a retransplant, call for a careful and personalized approach to patient management. Ongoing research and awareness initiatives are crucial for optimizing outcomes in these patients.
